# Integrative miRNA and Gene Expression Profiling Analysis of Human Quiescent Hepatic Stellate Cells

**DOI:** 10.1038/srep11549

**Published:** 2015-06-22

**Authors:** Mar Coll, Adil El Taghdouini, Luis Perea, Inge Mannaerts, Maria Vila-Casadesús, Delia Blaya, Daniel Rodrigo-Torres, Silvia Affò, Oriol Morales-Ibanez, Isabel Graupera, Juan José Lozano, Mustapha Najimi, Etienne Sokal, Joeri Lambrecht, Pere Ginès, Leo A. van Grunsven, Pau Sancho-Bru

**Affiliations:** 1 Institut d’Investigacions Biomèdiques August Pi i Sunyer (IDIBAPS), Barcelona, Spain; 2 Liver Cell Biology Lab, Faculty of Medicine and Pharmacy, Vrije Universiteit Brussel (VUB), Brussels, Belgium; 3 Centro de Investigación Biomédica en Red de Enfermedades Hepáticas y Digestivas (CIBERehd), Barcelona, Spain; 4 Laboratory of Pediatric Hepatology and Cell Therapy, Institute of Experimental & Clinical Research, Université Catholique de Louvain, Brussels, Belgium; 5 Liver Unit, Hospital Clínic, Faculty of Medicine, University of Barcelona, Barcelona, Spain

## Abstract

Unveiling the regulatory pathways maintaining hepatic stellate cells (HSC) in a quiescent (q) phenotype is essential to develop new therapeutic strategies to treat fibrogenic diseases. To uncover the miRNA-mRNA regulatory interactions in qHSCs, HSCs were FACS-sorted from healthy livers and activated HSCs (aHSCs) were generated *in vitro*. MiRNA Taqman array analysis showed HSCs expressed a low number of miRNAs (n = 259), from which 47 were down-regulated and 212 up-regulated upon activation. Computational integration of miRNA and gene expression profiles revealed that 66% of qHSC-associated miRNAs correlated with more than 6 altered target mRNAs (17,28 ± 10,7 targets/miRNA) whereas aHSC-associated miRNAs had an average of 1,49 targeted genes. Interestingly, interaction networks generated by miRNA-targeted genes in qHSCs were associated with key HSC activation processes. Next, selected miRNAs were validated in healthy and cirrhotic human livers and miR-192 was chosen for functional analysis. Down-regulation of miR-192 in HSCs was found to be an early event during fibrosis progression in mouse models of liver injury. Moreover, mimic assays for miR-192 in HSCs revealed its role in HSC activation, proliferation and migration. Together, these results uncover the importance of miRNAs in the maintenance of the qHSC phenotype and form the basis for understanding the regulatory networks in HSCs.

Quiescent hepatic stellate cells (qHSCs) are the major cell type responsible for the intrahepatic uptake, storage and secretion of vitamin A (retinoids). Moreover, they are the main regulators of the extracellular matrix (ECM) turnover within the space of Disse by expressing ECM proteins (mainly non-fibrillar ECM, collagen type III, IV and laminin) and by modulating the expression of metalloproteinases and their inhibitors. In response to fibrogenic stimuli HSCs activate and acquire a myofibroblast-like phenotype which is characterized by an aberrant production and deposition of ECM proteins potentially leading to liver fibrosis and resulting in the disruption of the vascular architecture^1^,^2^,^3^. While the cellular mechanisms underlying HSC activation have been extensively studied, the signals and regulatory pathways involved in the maintenance of HSC quiescence have barely been addressed. Moreover, most studies have been conducted in rodent models of liver disease and little information is available on human HSCs.

miRNAs are small non-coding RNAs of approximately 22 nucleotides that act as post-transcriptional gene expression regulators by degrading target mRNAs through perfect complementarity of the miRNA with the 3’ untranslated region (3’UTR) of mRNA or by inhibiting translation if an imperfect base pairing match exists^4^. Several miRNAs involved in HSC activation have been reported and their potential anti-fibrotic effects have been shown by inhibiting miRNAs function *in vivo* and *in vitro*^5^,^6^,^7^. One of the best studied miRNAs in HSCs is miR-21, which has been shown to be up-regulated in human and murine fibrotic liver samples. Indeed, knock down of miR-21 expression down-regulates the expression levels of *COL1A1* and *ACTA2* in primary HSCs. Furthermore, several miRNAs down-regulated during HSC activation have been suggested to play an anti-fibrogenic role^8^. That is the case for miR-150 and miR-194 among others, which are significantly down-regulated in HSCs isolated from bile duct-ligated (BDL) compared to sham operated rats. Moreover, several studies have shown a significant down-regulation of miR-29 in fibrotic mouse livers and miR-29b up-regulation was shown to repress HSC activation by regulating collagen synthesis in primary human HSC cultures^9^,^10^. Although a limited number of miRNAs have been described in both quiescent and activated HSCs (aHSCs)^8^,^9^,^10^,^11^,^12^, no global miRNA analysis has been performed on qHSCs. Thus far, little is known about miRNAs expressed in human qHSCs and their potential functional role in promoting the maintenance of quiescence.

By integrating miRNA and gene-expression data obtained through the analysis of human qHSCs and their respective culture-activated counterparts we identified relevant miRNAs involved in the maintenance of the quiescent phenotype of human HSCs. Moreover, the integrative analysis allowed us to identify miRNA-mRNA interaction networks potentially involved in HSC quiescence and activation. Finally, we have identified a panel of miRNAs with predicted target genes associated with HSC activation, and thus with a potential role in the repression of activation.

## Methods

### Patient samples

MiRNA and mRNA expression profiles were performed using HSCs and liver sinusoidal endothelial cells (LSECs) isolated from 4 cadaveric donors and 2 samples of hepatocytes obtained from two different donors. The protocol and conducted experiments were approved by the ethical committees of St-Luc Hospital and faculty of Medicine of Université Catholique de Louvain. An agreement from the Belgian Ministry of Health was obtained for the hepatocytes and hepatic stem cell bank. MiRNA expression validation was performed on liver samples of 14 healthy subjects and 15 patients with cirrhosis with superimposed alcoholic hepatitis (AH). The healthy controls were selected as previously described^13^. The protocol was approved by the ethics committee of the Hospital Clinic of Barcelona. Informed consents and the protocol conformed to the ethical guidelines of the 1975 Declaration of Helsinki were given and signed for all the patients. Clinical features of the cadaveric donors used for HSC isolation and cirrhotic patients are summarized in Supplementary Table 1 and 2, respectively.

### Isolation of high-purity qHSC and LSEC populations from healthy human livers

Human liver cells were isolated from the left liver segment of healthy donors up to 12 hours after clamping using a two-step perfusion technique^14^. Livers were kept on ice until sequential perfusion with an EGTA and digestion enzyme solution (0.9 mg/ml collagenase P and 0.03 mg/ml soybean trypsin inhibitor) was performed. Parenchymal cells were removed by low-speed (50 g) centrifugation steps. Non-parenchymal cells were suspended in a 5% FBS, 2 mM EDTA buffer and incubated for 30 minutes at 4°C with antibodies against CD32 (Abcam, Cambridge, United Kingdom) and CD45 (BD Biosciences, San Jose, CA) or with corresponding isotype controls. 7-aminoactinomycin D (eBioscience, San Diego, CA) was used to discriminate for non-viable cells. Enriched populations of human qHSCs were sorted out through a negative selection for CD32 (Ex: 488 nm; Em: 575 nm) and CD45 (Ex: 495 nm; Em: 519 nm) expressing cells and a positive selection for ultraviolet positivity (retinyl esters auto-fluorescence at 328 nm), using a fluorescence activated cell sorter (FACS-Aria BD Biosciences). Enriched populations of LSECs were obtained as CD32^+^CD45^−^ cells. Purified populations of qHSCs and LSECs were immediately used for total cell RNA and miRNA extraction.

### *In vitro* activation of human primary HSCs

Homogeneous populations of aHSCs were obtained as previously described^15^ by plating the cell fraction obtained by Nycodenz (Myegaard, Oslo, Norway) gradient centrifugation of the non-parenchymal cell fraction. Human aHSCs were cultured until passage four in Dulbecco’s modified Eagle’s medium (Lonza, Verviers, Belgium) supplemented with 10% fetal bovine serum (Biochrom GmbH, Berlin, Germany), at 37 °C in a humidified atmosphere with 5% CO_2_.

### Purity of human liver cell populations

The purity of the FACS sorted cell populations and culture-activated HSCs was assessed by quantitative real time PCR for HSC quiescence (*SPARCL1* and *ATP1B2*) and activation (*ACTA2, COL1A1 and LOX*) markers as well as the general HSC (*PDGFRB*), LSEC (*CD32b*), macrophage (*F4/80*) and hepatocyte (albumin) markers.

### Primary murine hepatic cells isolation

Male BALB/c mice (aged 20–25 weeks) were used and isolations were performed as described previously^16^,^17^. Briefly, after intraperitoneal anesthesia, the liver was perfused through the portal vein with buffers containing 0.25 mg/mL collagenase P (Roche Applied Science, Mannheim, Germany). Hepatocytes were separated from the non-parenchymal cell fraction by 2 min centrifugation at 50 g. Viable hepatocytes were further purified by gradient centrifugation on a 25% Percoll gradient (GE Healthcare Life Science, Diegem, Belgium). HSCs, LSECs and Kupffer cells (KCs) were collected from the non-parenchymal fraction by FACS (FACS Aria; Bectone Dickinson, Erembodegem, Belgium) using the endogenous UV-positivity in HSCs, a PE-coupled CD32 antibody (Invitrogen/molecular probes) for LSECs and an APC coupled F4/80 antibody for KCs (Life Technologies Corporation, Carlsbad, California, USA).

### Mouse models of liver fibrosis

Male BALB/c mice (aged 9–10 weeks) were used. The experimental procedures were approved by the institutional Animal Care and Use Committee of the Vrije Universiteit Brussel, permit number 12-212-2, and National Institutes of Health principles of laboratory animal care (NIH publication 86–23, revised 1995) were followed.

Chronic liver injury was induced by repeated intraperitoneal injections of carbon tetrachloride (CCl_4_ 50 μl /100 g body weight in mineral oil; both from Sigma) twice per week for 2 to 8 weeks or by common BDL (3–10 days). For common BDL, a peritoneal incision was made and the common bile duct was ligated and disrupted. Next, the abdominal cavity was closed with sutures and the animals were allowed to recover. At the end of the procedure, HSCs were isolated by FACS, based on UV-positivity. Isolated cells were not cultured, but immediately used for mRNA and miRNA analysis.

### miRNA and mRNA isolation and analysis

Total RNA containing miRNA was purified from qHSC and aHSC samples using RNeasy MICROKit (QIAGEN GmbH, Hilden, Germany) following a deviation of the standard protocol as recommended by the manufacturer and using 1.5 volumes of 100% ethanol before applying the sample into the column. Total RNA concentration and quality control was assessed using the RNA 6000 pico kit (Agilent, Santa Clara, CA).

Taqman human miRNA Array (card A and B) (Applied Biosystems, Life Technologies Corporation, Carlsbad, California, USA) was used to profile the expression of 758 miRNAs in 4 quiescent and 4 activated HSCs by PCR amplification and real time analysis. Each miRNA array was used in conjunction with pre-defined pools of primers for each array (Megaplex™RT Primers, Applied Biosystems). Due to the small quantity of starting material (30 ng each sample), miRNA cDNA samples were pre-amplified by PCR (Megaplex PreAmp Primers, Applied Biosystems) prior to loading on the array. The abundance of each miRNA in a total RNA sample was normalized to the level of the RNU6 expression (computation of –ΔCt). Fold changes were computed from the means of each group and statistical significance was established at p ≤ 0.05 (student t-test). A total of 13 mRNA samples, 4 qHSC together with their respective activated HSC samples, 2 hepatocyte samples and 3 LSEC samples were analyzed for gene expression profiling using Affymetrix HG-U219 genechips (Affymetrix, Santa Clara, California, USA). Raw data are made publically available on the NCBI Gene Expression Omnibus database, with accession number GSE67664.

### Integrative analysis of miRNA and mRNA expression data

In order to identify potential functional interactions of miRNAs with their target genes, we integrated gene and miRNA expression profiles obtained from both miRNA and mRNA arrays by using miRComb-R package ( http://mircomb.sourceforge.net). This software package allowed us to identify potential relevant miRNAs regulating target genes differentially expressed in qHSCs by first identifying differentially expressed genes and microRNAs, secondly calculating significant microRNA and mRNA correlations and third screening databases for known and predicted miRNA-mRNA target pairs. The first step consisted in the identification of differentially expressed genes and miRNAs in qHSCs.

We defined highly enriched mRNAs in qHSCs or “qHSC specific” genes as those with at least two-fold higher expression when comparing qHSCs to hepatocytes and LSECs. In addition, we refined this collection, by selecting only those mRNAs that are down-regulated upon HSC activation (FC qHSC vs aHSC ≥ 1.5).

Secondly, we classified mRNAs more highly expressed in aHSCs by using a cut-off of 2-fold higher expression than in hepatocytes and LSECs; again the number of genes in this category was reduced by selecting only those mRNAs that are significantly upregulated between qHSCs and aHSCs.

For the miRNAs we also used the 2-FC criteria for selecting those that are up-regulated during culture induced activation and a 1.5-FC cut-off was used for the miRNAs down-regulated during HSC activation.

In a second phase of the integration procedure we identified those miRNA-gene pairs showing negative correlation between miRNA and mRNAs. To accomplish this, Pearson correlation of the expressions of all possible combinations of deregulated mRNAs vs deregulated miRNAs were computed. Multiple testing correction was performed in order to reduce the number of false positive correlations and a final cut-off was set to FDR (false discovery rate) <0.05. Computed correlations are shown in Supplementary Figure 1. The third step in the integration procedure was to identify miRNA-mRNA pairs that were found to be predicted in the MicroCosm database. Basically, the package assigned a p-value to each miRNA-mRNA pairs according to the MicroCosm database (p_database) which predicts mRNA-miRNA interactions based on miRNA and mRNA 3’UTR alignment sequences^18^. Finally, only those pairs with significant negative correlation (FDR <0.05 and Pearson Coefficient <0) and predicted by the MicroCosm database as potential target (p_database <0.05) were selected as potential functional miRNA-mRNA interactions.

### Quantitative real time PCR (miRNA and mRNA)

Expression of mature miRNAs was assessed by reverse transcription using Mir-X miRNA First-strand Synthesis Kit (Takara, Dalian, China) according to the manufacturer’s instructions and qPCR reaction was performed by using SYBR^®^ Advantage qPCR premix (Takara). mRNA levels were determined by quantitative real time PCR on an ABI 7900HT cycler (Applied Biosystems) using SYBR green master mix (Life technologies). Individual gene and miRNA expression was normalized to GAPDH or U6 expression, respectively. Relative expression was calculated using the comparative Ct method (2^−ΔΔCt^). Gene specific primers were produced by Integrated DNA Technologies (Leuven, Belgium). Primer sequences used are listed in Supplementary Table 3.

### *In vitro* modulation of miRNA expression in human HSCs (LX2)

In order to validate relevant miRNA-mRNA interactions resulting from the integrative analysis, miR-21 and miR-100 expression were knocked down and miR-192 was over-expressed in a human HSC cell line (LX2) (kindly provided by Dr. Friedman). LX2 cells were grown in DMEM containing 10% FBS at 37 °C with 5% CO_2_ and humidity. LX2 cells were transfected with 50 nM of *mir*Vana^TM^ miRNA Inhibitor for miR-21 and miR-100 and *mir*Vana^TM^ miRNA mimic for miR-192 (Life Technologies) using JetPRIME® (PolyPlus, Illkirch, France) according to the manufacturer’s recommendations. *mir*Vana^TM^miRNA mimic and *mir*Vana^TM^miRNA inhibitor negative control (Life Technologies) were used as a calibrator sample to evaluate miRNA and target gene relative expressions. 24 hours after transfection, cells were harvested and immediately used for miRNA and mRNA expression analysis.

### Cell proliferation assay

Cell proliferation was assessed in mouse HSCs after 24 hours mimic miR-192 transfection (Life Technologies). Cell proliferation was measured as active DNA synthesis with the Click-iT EdU Cell Proliferation Assay Kit (Invitrogen, Eugene, OR), following the manufacturer’s instructions as described before^19^. In brief, 7500 cells were seeded per cm^2^, 48 hours after miRNA mimic transfection cells were incubated with EdU for 48 hours prior to fixation and staining of the cells at day 5 of the culture. The ratio of total cells and EdU-incorporated cells was calculated.

### Cell migration assay

Cell migration of mouse primary HSCs was determined by a trans-well migration assay as described before^20^, using PDGF-BB (20 ng/ml) as a chemoattractant. In brief, cells were transfected with 50 nM miRNA mimic at day 5 and day 7 of the culture. At day 8, 100.000 cells were transferred to a collagen I-coated transwell chamber. One hour later, PDGF-BB was added to the lower compartment and cells were allowed to migrate for 18 hours.

### Cell activation assay

Freshly isolated, primary mouse HSCs were seeded at a density of 15.000 cells/cm2 and transfected with miR-192 or control mimic 24 h after plating. On day 2, transfected cells were serum-starved overnight and subsequently exposed to 10 ng/ml human recombinant TGFβ1 (R&D) for 48 h, prior to being lyzed for RNA extraction.

### Statistical analysis

GraphPad Prism v4.0.0 (GraphPad Software, La Jolla, CA) and SPSS V.14.0 for Windows (SPSS Inc, Chicago, Illinois, USA) were used for statistical analysis. Data in the figures are expressed as means ± SEM. Differences among groups were tested for statistical significance by Student t-test or Mann Whitney U test when appropriate. Ns = not significant p ≥0.05, *p <0.05, **p <0.01, ***p < 0.001.

## Results

### Characterization of FACS-sorted human liver cell populations and culture activated HSCs

**Figure 1:**
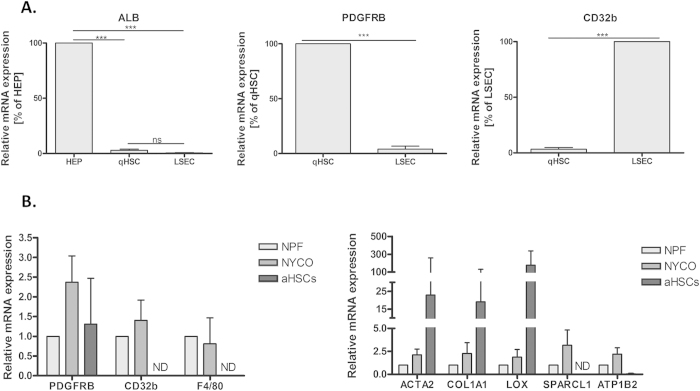
Purity of sorted cell populations and cultured hepatic stellate cells: (**A**) Relative expression of hepatocyte (*albumin*), HSC (*PDGFRB*) and LSEC (*CD32b*) markers in qHSC, LSEC and hepatocyte samples (n = 3; each group). (**B**) Relative expression of liver cell markers (*PDGFRB*, *CD32b* and *F4/80*), activation markers (*ACTA2, COL1A1, and LOX*) together with quiescent markers (*SPARCL1, ATP1B2*) in non-parenchymal fraction (NFP), nycodenz qHSC fraction (NYCO) and aHSCs (passage 4).

Human liver cells were isolated from healthy liver donors. Hepatocytes were isolated by enzymatic perfusion of the liver and low-speed centrifugation steps in two different donors. Enriched populations of qHSCs (UV^+^CD32^−^CD45^−^) and LSECs (CD32^+^CD45^−^) were purified from the resulting non-parenchymal cell fraction by FACS-sorting. Moreover, qHSCs isolated by density gradient from the same patients were cultured until passage four to obtain pure populations of aHSCs. To assess the purity of the different cell populations we measured the expression of specific markers for each liver cell type. qHSC and LSEC sorted fractions were highly enriched in *PDGFRB* and *CD32b* respectively and no significant expression of albumin was detected (Fig. 1A). Purity and activation of the *in vitro* activated HSCs were assessed by measuring the expression levels of classical activation and quiescent markers in the non-purified non-parenchymal cell fraction (NPF), in the qHSC-enriched nycodenz fraction and in culture-activated HSCs (Fig. 1). Culture-activated HSCs expressed *PDGFRB* but not *CD32b* or *F4/80* indicating the absence of contaminating LSECs and KCs respectively. In addition, aHSCs expressed high levels of the classical activation markers *ACTA2*, *COL1A1* and *LOX* and low expression levels of *SPARCL1* and *ATP1B2* compared to non-parenchymal cells and the qHSC enriched nycodenz fraction (Fig. 1B).

### MiRNA profiling of human quiescent HSCs

**Figure 2:**
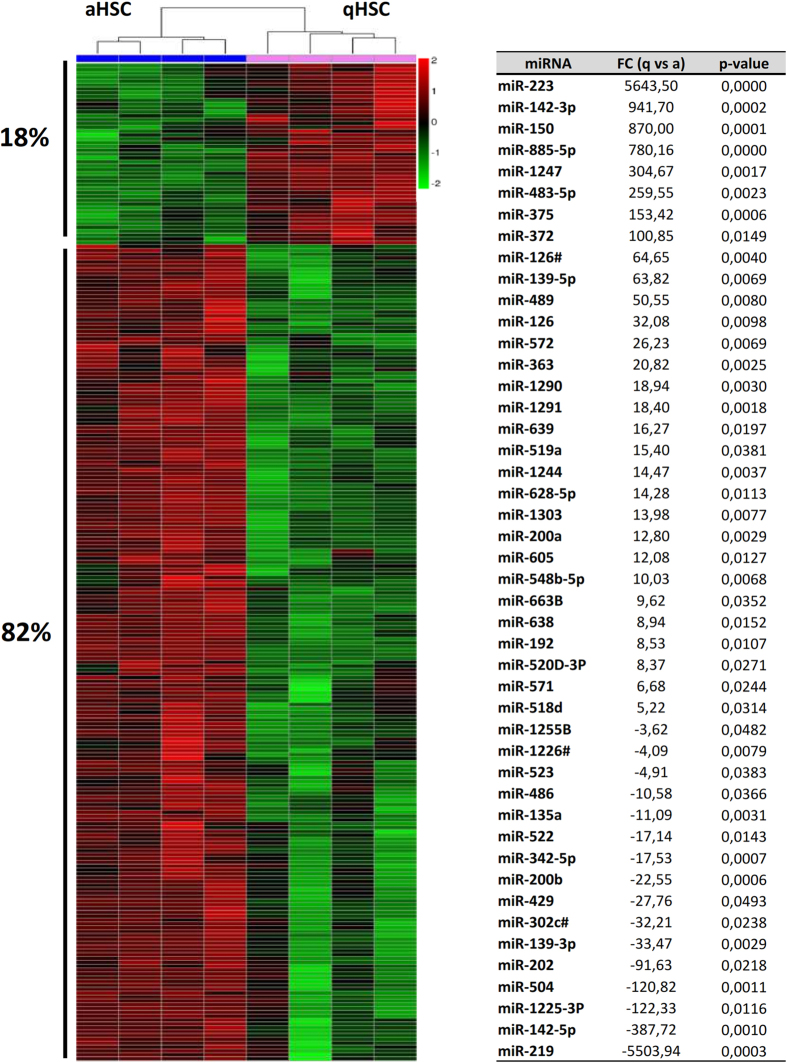
MiRNA expression profile of human quiescent HSCs. Heat map showing the differentially expressed miRNAs in freshly isolated qHSCs compared to their respective culture-activated HSCs (n = 4 each group). miRNA expression data was generated by performing Taqman human miRNA Array. The intensity of each colour indicates the standardized ratio between intensity and the average expression of each miRNA across all samples. Red colour pixels indicate an increased abundance of miRNA in the indicated samples whereas green pixels indicate decreased miRNA levels in Log2 scale. The top miRNAs significantly deregulated in human quiescent compared to *in vitro* activated HSC are summarized in the table.

In order to determine the miRNA expression profile of qHSCs we compared the expression levels of 758 miRNAs in four qHSC samples and their corresponding *in vitro* activated cells. Surprisingly, among the differentially expressed miRNAs only 47 (18%) were found to be highly expressed in qHSCs and significantly down regulated upon *in vitro* activation (p < 0.05). In contrast, 212 (82%) miRNAs barely expressed in qHSCs were significantly up-regulated during activation (Fig. 2). The top significantly up-regulated miRNAs upon activation are summarized in Supplementary Table 4.

### Integrative miRNA-mRNA expression analysis

**Figure 3:**
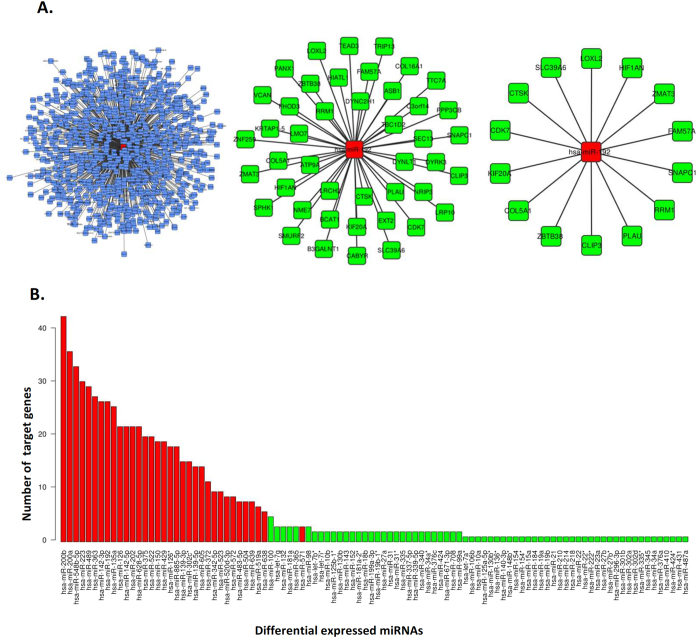
MiRNA-mRNA integrative analysis. (**A**) miRNA-mRNA integration analysis workflow. The diagram shows the reduction of the potential mRNA targets for miR-192 achieved by integrating miRNA and gene expression data: first, miR-192 is connected with its targets predicted by the Microcosm database; second, miR-192 is connected with its targets predicted by Microcosm and also found deregulated during activation of human HSCs (p < 0.01) and finally, the functionally relevant miR-192 targets based on significant negative correlation between miR-192 and its predicted targets and the MicroCosm prediction value is shown (**B**) Histogram representing differentially expressed miRNAs according to the number of deregulated target genes assigned by miRNA-gene integration analysis (p < 0.01). Red bars indicate miRNAs highly expressed in qHSCs whereas green bars show miRNAs highly expressed in activated HSC.

To identify deregulated miRNAs with a potential role during HSC activation we assessed miRNA expression profiles in the context of the expression of their target genes (Fig. 3A). Integration of miRNA and gene expression profiles from human qHSCs and aHSCs revealed a set of miRNAs that negatively correlated with the expression of their predicted target genes. Interestingly, when we plot the differentially expressed miRNAs with the number of their differentially expressed predicted target genes we observed that the miRNAs expressed in qHSCs cluster together due to the large number of potentially regulated target genes (Fig. 3B). In contrast, the integrative analysis assigned a smaller number of target genes to the miRNAs up-regulated in the activated phenotype (Fig. 3B). Indeed, 66% of miRNAs expressed in qHSCs were associated with at least 6 target genes with an average of 17,28 ± 10,7 target genes per miRNA, whereas we did not find any miRNA up regulated in aHSCs with more than 4 predicted targets except for miR-100 (Fig. 3B). Activated HSC-associated miRNAs had an average of 1,49 ± 0,7 target genes. These results indicate that a relatively small number of miRNAs potentially regulates an important number of genes in qHSCs, while the opposite (a large number of miRNAs each of them regulating a small number of genes) may be taking place in activated cells.

In order to investigate whether miRNAs expressed in qHSCs were targeting/inhibiting the activation process we performed an *in silico* functional analysis with Ingenuity Pathway Analysis® software. As we expected, the interaction networks generated with genes targeted by miRNAs expressed in qHSCs were significantly related to key activation processes such as cellular movement, growth and proliferation as well as cell morphology. Moreover, hepatic fibrosis and HSC activation (p-value = 4.36·10^−6^) as well as actin cytoskeleton signaling (p-value = 0.017) pathways were found enriched in this set of target genes. These results indicate that qHSCs express a set of miRNAs that may be responsible, at least in part, for maintaining the quiescent phenotype by repressing genes involved in HSC activation. Table 1 shows the miRNAs that are highly expressed in quiescent HSCs and have a high number (n > 6) of predicted target genes.

### Expression of miRNAs in human liver tissue

**Figure 4:**
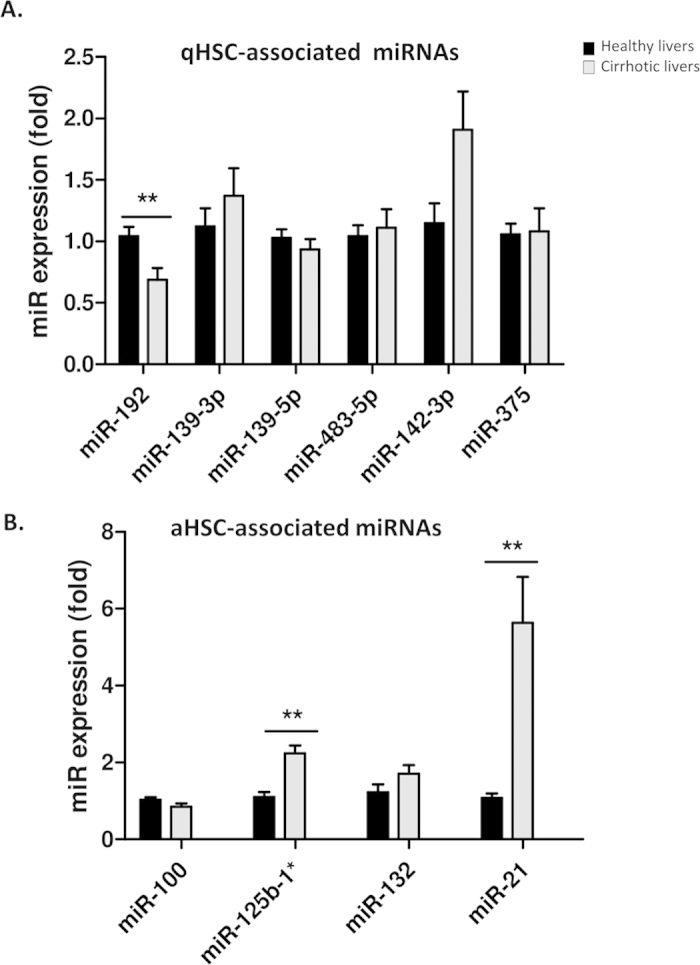
MiRNA expression levels in cirrhotic and healthy human livers. (**A**) Expression levels of qHSC enriched miRNAs in cirrhotic (n = 14) and healthy (n = 15) human liver samples. (**B**) Expression levels of aHSC enriched miRNAs in cirrhotic (n = 15) and healthy (n = 14) human liver samples.

Although HSCs are the major cell type responsible for liver fibrogenesis, they represent only 8% of the total liver cells. In order to explore if miRNAs dysregulated in HSCs could be detected in human liver we assessed the expression of selected miRNAs in cirrhotic (n = 15) and healthy (n = 14) human liver tissues. MiR-192 was found significantly down regulated (relative expression of 0.68, fold change = −1.47) in cirrhotic livers compared to healthy livers indicating that this miRNA, which we have identified as highly expressed in qHSC by the miRNA array, could also be detected in healthy tissue (Fig. 4A). On the other hand, miR-125b-1* and miR-21 significantly increase their expression in cirrhotic liver compared to healthy tissue (fold change = 2.2 and 5.6, respectively) (Fig. 4B).

### *In vitro* modulation of miR-192 in activated human HSCs

**Figure 5:**
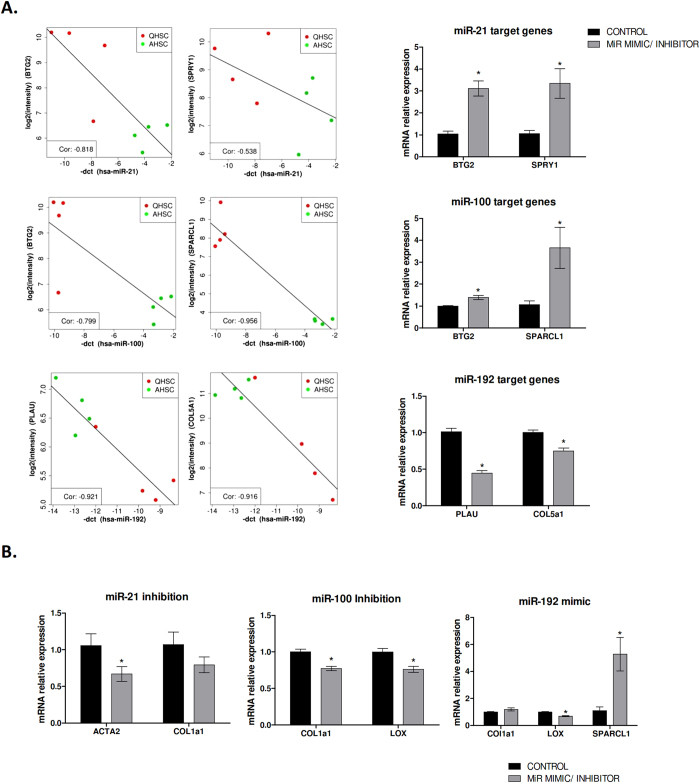
***In vitro*** modulation of miRNA-21, miRNA-100 and miRNA-192 expression in LX2 cells. Reduction of miR-21 and miR-100 expression and up-regulation of miR-192 in LX2 cells was achieved by transfecting miR-21 antagomir (50 nM), miR-100 antagomir (50 nM) or miR-192 mimic (50 nM), respectively (n = 3). (**A**) Box plots show the significant negative correlation between miRNA and target gene expressions. MiR-21 and miR-100 inhibition causes an increase of the expression levels of their target genes while miR-192 overexpression decreases target gene expression levels (**B**) *In vitro* modulation of miR-21, miR-100 and miR-192 expression result in a reduction of HSC activation.

**Figure 6:**
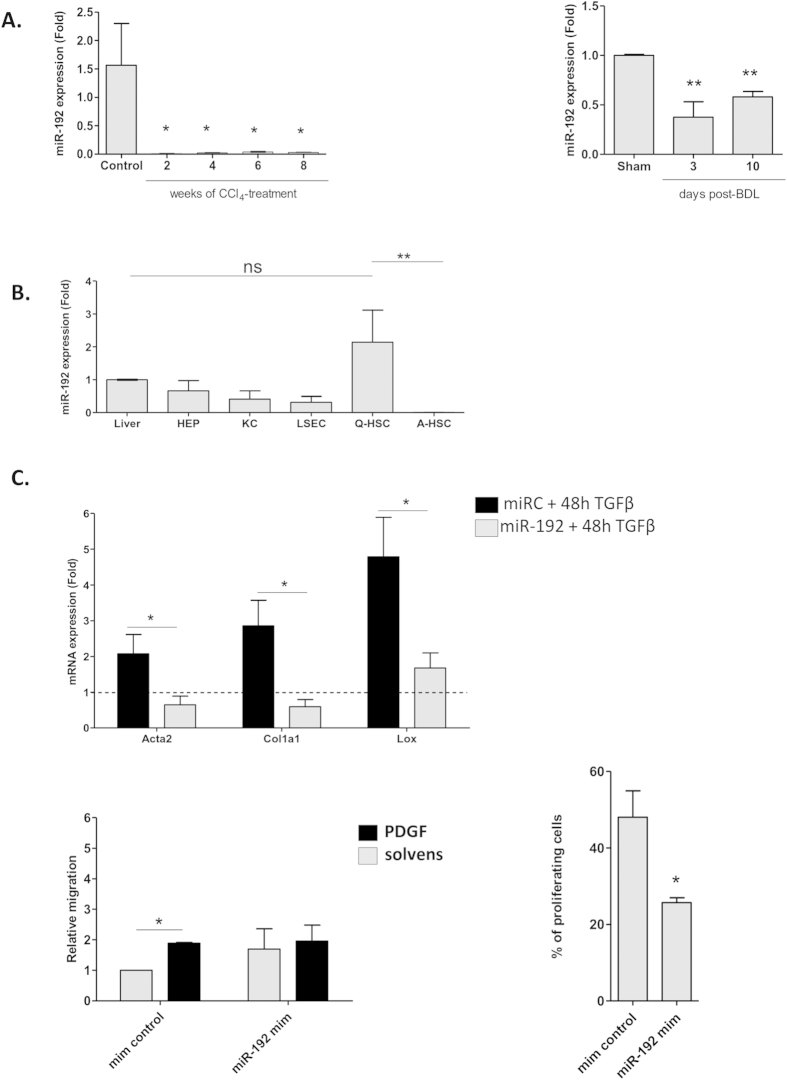
Expression and function of miR-192 in primary mouse HSCs. (**A**) Expression of miR-192 in mouse HSCs during *in vivo* activation. Mir-192 expression levels were assessed in HSCs isolated from control mice, mice treated with CCl_4_ and from mice after BDL- or sham-operation at indicated time points after treatment or surgery. miRNA expression is shown relative to control or sham operated mice. (**B**) miR-192 expression in different hepatic cell types isolated from healthy mouse livers. MiR-192 expression was evaluated by qPCR in hepatocytes, quiescent and *in vitro* activated HSCs, KCs and LSECs. Mir-192 expression is shown relative to whole liver (**C**) Effect of miR-192 over-expression on cell activation, migration and proliferation in primary mouse HSCs. The contribution of miR-192 to HSC proliferation was evaluated in transfected mouse HSCs 1 day after miR-192 mimic transfection. HSCs were exposed to EdU, fixed 2 days later and stained for DNA-incorporated EdU. Proliferation is represented by the percentage of EdU-positive cells. Contribution of miR-192 on mouse HSC migration was assessed by a transwell migration assay on primary mouse HSCs transfected with mimic miR-192. PDGFbb (20 ng/mL) was added to promote cell migration. After 18 hours migrated cells were stained with DAPI and counted. Results are represented as migration relative to the non-PDGF stimulated cells. Finally, the effect of overexpressing miR-192 on cell activation was evaluated by exposing freshly isolated mice HSCs to TGFβ1 (10 ng/mL) or solvent for 48 hours. Results are represented as relative expression to miR control incubated with solvent.

MiR-192 has a high number of predicted target genes (n = 28) in qHSCs, displays a significant reduction in expression between healthy and cirrhotic human liver tissue and it has not been previously reported to be associated with HSC or liver fibrosis. For those reasons, we selected miR-192 for further functional characterization by modulating its expression in human and mouse HSCs (Figs 5 and 6). As a control miRNA we used miR-100 and miR-21. MiR-100 is the up-regulated miRNA following HSC activation and has the highest number of predicted targets and miR-21 is not only up-regulated in activated HSC but also in human cirrhotic tissue.

As expected, by increasing the expression levels of miR-192 we observed a significant reduction in the expression of its target genes, *PLAU* and *COL5A1*, as well as a modest but significant reduction of the activation marker *LOX* and an increase in *SPARCL1*, a novel quiescence marker identified in the gene expression array. On the contrary, an inhibition of the endogenous miR-100 expression level enhanced the expression of its two predicted target genes, i.e. *BTG2* and *SPARCL1*, but also caused a ~20% reduction of the basal expression of pro-fibrogenic genes such as *COL1A1* and *LOX*. Accordingly, miR-21 inhibition resulted in the reduction of miR-21 target genes expression (*BTG2* and *SPRY1*) and also in a significant decrease of *ACTA2* (Fig. 5A,B). Altogether, these results validate the computational integrative analysis and on the other hand they confirm the role of these microRNAs as regulators of the aHSC phenotype by modulating the expression of activation markers.

### MiR-192 is highly expressed in qHSCs and might play a functional role in suppressing HSC activation

Next, to understand the dynamics of miR-192 expression during HSC activation, we used HSCs isolated from mouse livers. MiR-192 expression was evaluated in HSCs isolated at different time points from two different fibrotic mouse models: bile duct ligation surgery (BDL) and chronic CCl_4_ administration. Interestingly, miR-192 expression in HSCs was clearly reduced during fibrogenesis at very early time points in both animal models indicating that down-regulation of miR-192 in HSCs might be an early event during HSC activation and fibrogenesis (Fig. 6A). In order to identify other cells producing miR-192 in the healthy liver, miR-192 expression was assessed in freshly isolated hepatic cell populations (i.e. hepatocytes, KCs, LSECs and qHSC) from healthy mouse livers. Our results indicate that qHSCs are the major source of miR-192 expression in the healthy liver and that the expression of this miRNA in HSCs is lost upon culture-induced and *in vivo* activation (Fig. 6B). Additionally, we aimed to explore whether the level of miR-192 functionally contributed to the reduction of HSC activation (Fig. 6C). We first evaluated the effect of miR-192 in primary mouse qHSCs. Importantly, miR-192 overexpression clearly inhibited the TGFβ1-induced activation of qHSCs as measured by the suppression of *Acta2*, *Col1a1* and *Lox* expression, three key early activation markers in HSCs (Fig. 6C). Next we tested if miR-192 was able to repress functional activation features. Transfection of primary mouse aHSCs with miR-192 mimics showed a marked (42.7 ± 0.11%) reduction in cell proliferation compared to cells that were transfected with the control mimic. Consistent with this result, PDGF-induced migration of mouse HSCs was reduced by miR-192 transfection (Fig. 6C). Taken together these results suggest that miR-192 is a qHSC enriched miRNA in the liver with a functional role during HSC activation.

## Discussion

In the present study we provide the first miRNA expression profile in human HSCs. Moreover, to identify key miRNAs involved in the maintenance of the quiescent phenotype and explore the pathways that might be regulated by miRNA, we have combined gene and miRNA expression data from human qHSCs and from corresponding aHSCs. Our study identified a reduced panel of miRNAs highly expressed in qHSCs which are predicted to target a high number of genes known to be involved in the activation process. Moreover, this study provides basic information on miRNAs in HSCs and forms the basis for further studies deciphering the role of miRNAs in liver fibrosis.

HSCs have been extensively studied due to their important role during liver fibrogenesis^21^,^22^,^23^. However, most studies have been performed with rodent HSCs and human information is still limited. Studies on qHSCs have been hampered by the lack of good methods to maintain HSCs quiescent in culture, even for a short period of time. Indeed, a number of studies have shown that the gene expression pattern of HSCs is altered after a few hours in culture^24^. Actually, culture-activated human HSCs acquire a phenotype that, although not identical, resembles the activation state of HSCs in the fibrotic liver^25^. In this study we have assessed the gene and miRNA expression profile of freshly un-cultured human qHSCs. Cells were isolated through cell sorting by both immunoselection and UV auto-fluorescence. It is important to notice the novelty of this procedure, since it varies substantially in terms of isolation conditions and phenotype of the cells from already described procedures with mouse cells using FACS sorting approaches^19^,^26^. To be able to identify which miRNAs changed their expression during cell activation, miRNA and gene expression profiling was performed in cells activated *in vitro* from the same individuals from which quiescent cells were isolated. The use of culture-activated HSCs allowed us to minimize the variability existing among patients with chronic liver diseases due to etiology, stage of disease development or sample collection procedure.

The expression level of a miRNA provides limited information about its role. miRNAs are non-coding regulators of gene expression and therefore, their role depends on their level of expression but also the expression of their target genes^27^,^28^. Since the role of a miRNA can only be defined if we know its spatiotemporal coexistence with its target mRNA, it was important to assess the miRNA expression profile in the context of the expression of their target genes in both human qHSCs and aHSCs. Moreover, we have used the miRComb-R package, an integrative bioinformatics tool, able to predict potential biologically relevant miRNA-mRNA interactions based on both miRNA-mRNA expression correlations and sequence hybridization predicted in well-known databases^12^,^29^. This integrative approach allowed us to identify miRNAs not previously associated with HSC biology, but also to identify new targets for miRNAs already described to play a role in HSCs and fibrosis.

An interesting observation of the present study is the consistent up-regulation of miRNAs during *in vitro* HSC activation. Actually, approximately 80% of the differentially expressed miRNAs (212/259) were found expressed at low levels in the qHSCs and up-regulated in aHSCs. By contrast, the transcriptome analysis showed a more balanced ratio of up and down-regulated genes during activation. In order to understand the relevance of these findings, we merged the miRNA and gene expression profiles to reveal the potential miRNA-target interactions.

One of the most important findings of this study is the higher number of target genes assigned by the computational integrative analysis to miRNAs expressed in quiescent cells compared to those measured in their activated counterparts. Importantly, miRNAs expressed in qHSCs targeted genes involved in HSC activation and liver fibrosis such as different collagen types (*COL5A1,COL4A5, COL8A1, COL9A3*), *LOX*, *TGFβ* or *IL-6*^1^,^20^,^30^. These results were further supported by the *in silico* functional analysis of these targeted mRNAs showing their association with important pathways regulating HSC activation. These results may indicate that miRNAs highly expressed in qHSCs could be acting as suppressors of HSC activation, thereby preventing HSC activation towards myofibroblast-like cells. This would suggest that HSC quiescence is an actively maintained state. This finding has a potential physiological and clinical relevance as it suggests that strategies aiming at preventing HSC activation could be based on mimicking the function of these miRNAs expressed in qHSCs.

Among all miRNAs expressed in qHSCs, we have highlighted a subset of 31 miRNAs which are targeting/inhibiting more than 6 up-regulated genes during activation. It is important to notice that these 31 miRNAs are targeting 27% (267 out of 998) of all up-regulated genes during activation and some of these miRNAs have been previously reported to be associated with activation of HSCs. For instance, miR-150 has been shown to be down-regulated in HSCs isolated from rat fibrotic livers^12^, while expression of miR-126 and miR-126* were reduced during *in vitro* and *in vivo* activation of rat HSCs^31^. Other miRNAs such as miR-192, miR-139-5p, miR-483-5p, miR-142-3p, miR-142-5p, or miR-375 have not been previously described to be expressed in HSCs.

Among all miRNAs identified in qHSCs, miR-192 was selected for further functional assays in human and murine HSCs. The selection was based on its enrichment in quiescent HSC and healthy liver tissue, the high number of predicted targets, the loss of expression in cirrhotic patients as well as the novelty of miR-192 in the context of liver fibrosis. We first studied whether miR-192 could be considered as a quiescent-specific miRNA during both *in vitro* and *in vivo* activation. As found in human, HSCs derived from healthy mice presented significant higher levels of miR-192 compared to the HSCs isolated from fibrotic mice and its expression was rapidly reduced upon induction of fibrosis *in vivo*. In the present study, we also provide evidence for the role of miR-192 in the regulation of HSC activation by showing that overexpression of miR-192 suppresses TGFβ1-induced up-regulation of classical activation genes, i.e*. Acta2*, *Col1a1* and *Lox*. Additionally, we find that miR-192 overexpression significantly repressed the proliferation and migratory potential of primary mouse HSCs, both key functional properties acquired during HSC activation. Moreover, miR-192 mimics reduced the expression of key activation markers in a human HSC cell line. Although the modulation of individual activation markers by miR-192 mimic may be modest, we provide evidence that miR-192 has a functional effect on HSCs. Particularly, these findings indicate that miR-192 can actively promote a quiescent phenotype in fibrogenic conditions and suggest potential therapeutic value for the modulation of miR-192 expression.

Our data also provides information regarding the miRNAs that may be playing a role during HSC activation. Interestingly, the bioinformatic analysis assigned a small number of targets to the miRNAs up-regulated in activated state. Among all miRNAs found highly expressed in aHSCs, miR-100 is the miRNA with the highest number of predicted target genes. Interestingly, according to the mRNA-miRNA integrative analysis performed, SPARCL1-like (*SPARCL1*) is the gene that is targeted by the highest number of miRNAs (10 miRNAs up-regulated in aHSC are predicted to target *SPARCL1*) (Supplementary Table 5). Moreover, when we analyzed the transcriptomic data obtained by comparing activated *vs* quiescent HSC gene expression we found that *SPARCL1* was strongly (fold change = −40) downregulated during HSC activation and this infra-expression was also validated by qPCR. SPARCL1 is a protein member of the matricellular SPARC family of proteins, a diverse group of proteins that modulate cell interaction with the extracellular milieu. SPARCL1 is the only SPARC family member, in addition to SPARC itself, which can bind to fibrilar collagens and influence collagen fibril architecture^32^,^33^. In concordance with the integrative analysis, functional *in vitro* assays showed that mir-100 inhibition resulted in *SPARCL1* up-regulation and in a significant reduction of *COL1A1* and *LOX* expression levels.

In conclusion, by integrating miRNA and mRNA expression data obtained from quiescent and activated human HSCs we have identified a panel of miRNAs highly expressed in qHSCs compared to aHSCs. These miRNAs are predicted to target over 25% of all up-regulated genes following HSC activation and therefore might have a common functional role in repressing HSC activation. Among all miRNAs included in this panel we have shown that miR-192 is down-regulated both during *in vivo* and *in vitro* activation of qHSCs and that overexpression of miR-192 exerts a functional role during the activation program by reducing activation, migration and proliferation of HSCs. Further studies are needed to evaluate whether the identified miRNAs have a coordinated functional role in the maintenance of the quiescent phenotype and the repression of HSC activation.

## Additional Information

**How to cite this article**: Coll, M. *et al.* Integrative Mirna and Gene Expression Profiling Analysis of Human Quiescent Hepatic Stellate Cells. *Sci. Rep.* **5**, 11549; doi: 10.1038/srep11549 (2015).

## Supplementary Material

Supplementary Information

## Figures and Tables

**Table 1:** Panel of miRNAs enriched in qHSCs with the number of deregulated predicted target genes.

**Quiescent miRNAs**			
**miRNA**	**FC (Q vs. A)**	**p-value**	**#target genes**
**miR-200b**	22,55	0,0006	45
**miR-200a**	12,8	0,0029	38
**miR-548b-5p**	10,03	0,0068	35
**miR-223**	5643,5	2.06·10–5	32
**miR-489**	50,55	0,008	31
**miR-363**	20,82	0,0025	29
**miR-142-3p**	941,7	0,0002	28
**miR-192**	8,53	0,0107	28
**miR-135a**	11,09	0,0031	27
**miR-126**	32,08	0,0098	23
**miR-142-5p**	387,72	0,001	23
**miR-202**	91,63	0,0218	23
**miR-628-5p**	14,28	0,0113	23
**miR-375**	153,42	0,0006	21
**miR-522**	17,14	0,0143	21
**miR-150**	870	0,0001	20
**miR-429**	27,76	0,0493	20
miR-126*	64,65	0,004	19
**miR-885-5p**	780,16	4,34·10–6	19
**miR-139-3p**	33,47	0,0029	16
miR-302c*	32,21	0,0238	16
**miR-139-5p**	63,82	0,0069	15
**miR-605**	12,08	0,0127	15
**miR-372**	100,85	0,0149	12
**miR-342-5p**	17,53	0,0007	10
**miR-520d-3p**	8,37	0,0271	9
**miR-572**	26,23	0,0069	9
**miR-483-5p**	259,55	0,0023	8
**miR-504**	120,82	0,0011	8
**miR-639**	16,27	0,0197	8
**miR-519a**	15,4	0,0381	7
