# Supplementary material for: Integrative miRNA and Gene Expression Profiling Analysis of Human Quiescent Hepatic Stellate Cells
Source: Sci Rep. 2015 Jun 22;5:11549. doi: 10.1038/srep11549 (PMC4476106; doi:10.1038/srep11549)

# SUPPLEMENTARY INFORMATION

## INTEGRATIVE miRNA AND GENE EXPRESSION PROFILING ANALYSIS OF HUMAN QUIESCENT HEPATIC STELLATE CELLS

Mar Coll[1*], Adil El Taghdouini[2*], Luis Perea[1], Inge Mannaerts[2], Maria Vila-Casadesús[3], Delia Blaya[1], Daniel Rodrigo-Torres[1], Silvia Affò[1], Oriol Morales-Ibanez[1], Isabel Graupera, Juan José Lozano[3], Mustapha Najimi[4], Etienne Sokal[4], Joeri Lambrecht[2], Pere Ginès[1,3,5], Leo A. van Grunsven[2*] and Pau Sancho-Bru[1,3*].

**equal contribution*

1 Institut d'Investigacions Biomèdiques August Pi i Sunyer (IDIBAPS), Barcelona, Spain.

2 Liver Cell Biology Lab, Faculty of Medicine and Pharmacy, Vrije Universiteit Brussel (VUB), Brussels, Belgium.

3 Centro de Investigación Biomédica en Red de Enfermedades Hepáticas y Digestivas (CIBERehd), Barcelona, Spain.

4 Laboratory of Pediatric Hepatology and Cell Therapy, Institute of Experimental & Clinical Research, Université Catholique de Louvain, Brussels, Belgium.

5 Liver Unit, Hospital Clínic, Faculty of Medicine, University of Barcelona, Barcelona, Spain.

**Supplementary Table 1.**Clinical characteristics of cadaveric liver donors

| liver ID | isolated cells | age | gender | ischemia time | Cause of brain death |
|---|---|---|---|---|---|
| **L4** | qHSC and LSEC | 12 years | female | 16h30 | Drowned |
| **L8** | qHSC and LSEC | 1 days | male | 4h40 | Sudden death of unknown cause |
| **L10** | qHSC and LSEC | 7 months | female | 5h20 | Sudden death of unknown cause |
| **L11** | qHSC and LSEC | 7 days | male | 4h25 | Sudden death of unknown cause |
| **F3** | Hepatocytes | 17 years | male | 9h | Trauma |
| **F23** | Hepatocytes | 16 years | female | <10h | Trauma |

**Supplementary Table 2.** Characteristics of patients with cirrhosis and alcoholic hepatitis

| Validation Cohort (n=15) | |
|---|---|
| **Characteristics** | Median (25-75 IQR) |
| Age (y) | 54 (49-60) |
| Male n (%) | 87 |
| Alcohol intake (g/day) | 100 (90-200) |
| **Laboratory and hemodynamic parameters** | |
| Hemoglobin (g/dL) | 11.8 (10.7-13.5) |
| Leukocyte count $x10^9/L$ | 7.64 (4.3-11) |
| Platelet count $x10^9/L$ | 124 (84-208) |
| AST ( U/L) | 127 (104-240) |
| ALT (U/L) | 58 (14-67) |
| Serum albumin (g/dL) | 29 (24-32.5) |
| Serum creatinine (mg/dL) | 0.9 (0.6-1.3) |
| Serum bilirubin (mg/dL) | 10 (1.7-19.6) |
| International normalized ratio | 1.4 (1.2-1.8) |
| HVPG (mmHg) | 17 (10-22) |
| **Alcoholic hepatitis severity** | |
| MELD score | 21 (11-24) |
| ABIC score | 7.9 (6.8-8.8) |
| Maddrey score | 68.3 (62.8-82.5) |

IQR, interquartile range; AST, aspartate aminotransferase level; ALT, alanine aminotransferase level; HVPG, hepatic venous pressure gradient; MELD, model for end-stage liver disease; ABIC, age-bilirubin-INR-creatinine score.

**Supplementary Table 3.**List of primers used for gene expression analysis

| Gene Name | Forward sequence | Reverse sequence |
|---|---|---|
| **GAPDH** | AGCCACATCGCTCAGACAC | GCCCAATACGACCAAATCC |
| **ALBUMIN** | TGGCACAATGAAGTGGGTAA | CTGAGCAAAGGCAATCAACA |
| **PDGFRβ** | CCCTTATCATCCTCATCATGC | CCTTCCATCGGATCTCGTAA |
| **CD32b** | AGTGCCCAGCATGGGCAGC | TGTCTCTTTCTGATGGCAAT |
| **F4/80** | GCCAGTTTGCACAGTTATAGTGA | GACTTCGGCATTAACACTGGA |
| **ACTA2** | CTGTTCCAGCCATCCTTCAT | TCATGATGCTGTTGTAGGTGG |
| **COL1A1** | GACACAGAGGTTTCAGTGG | CACCCTTAGCACCAACAG |
| **LOX** | CACAGTTGTCATCAACATTACC | CTGACCTTTAGGATATAGTTTCCAG |
| **SPARCL1** | GGATTCAACTTCAATTTTTCTGC | AGAAGGTTGGTTGGATTACTTGA |
| **ATP1B2** | GAGGACGCACCAGTTTATGG | AACATGGCGGTGAGGAAC |
| **BTG2** | AGCGAGCAGAGGCTTAAGGT | CGGTAGGACACCTCATAGGG |
| **SPRY1** | GTGTGTTGGAAATCCACG GT | AAAGAAGGCTGCTGGATCAC |
| **PLAU** | TCACCACAACGACATTGCCTT | TGATCTCACAGCTTGTGCCAAA |
| **COL5A1** | CACAACTTGCCTGATGGAATAACA | GCAGGGTACAGCTGCTTGGT |
| **miRNA** | **primer sequence** | |
| **hsa-miR-192** | CTGACCTATGAATTGACAGCC | |
| **mmu-miR-192** | CTGACCTATGAATTGACAGCC | |
| **hsa-miR-139-5p** | TCTACAGTGCACGTGTCTCCAGT | |
| **hsa-miR-139-3p** | TGGAGACGCGGCCCTGTTGGAGT | |
| **hsa-miR-485-5p** | AAGACGGGAGGAAAGAAGGGAG | |
| **hsa-miR-142-3p** | TGTAGTGTTTCCTACTTTATGGA | |
| **hsa-miR-375** | TTTGTTCGTTCGGCTCGCGTGA | |
| **hsa-miR-100** | AACCCGTAGATCCGAACTTGTG | |
| **hsa-miR-125b-1*** | ACGGGTTAGGCTCTTGGGAGCT | |
| **hsa-miR-132** | TAACAGTCTACAGCCATGGTCG | |
| **hsa-miR-21** | UAGCUUAUCAGACUGAUGUUGA | |

**Supplementary Table 4.**Up-regulated miRNAs in human culture-activated compared to quiescent HSCs.

| miRNA | FC (Q vs. A) | P value | Target genes |
|---|---|---|---|
| **miR-100** | 112,49 | $6{\cdot}10^{-7}$ | CPNE5, BTG2, SPARCL1, PPAP2A, ABCG2 |
| **miR-99a** | 74,36 | $1{\cdot}10^{-6}$ | CPNE5, BTG2 |
| **miR-130b** | 11,38 | $6{,}3{\cdot}10\text{-}^{5}$ | ITGB4, ENTPD1 |
| **miR-625** | 7,93 | 0,0003 | C7 |
| **miR-29a** | 68,21 | 0,0003 | none |
| **miR-222** | 37,30 | 0,0003 | none |
| **miR-28** | 6,39 | 0,0003 | none |
| **miR-365** | 8,56 | 0,0005 | ST8SIA6, NR4A2, RASD1 |
| **miR-576-3p** | 15,96 | 0,0006 | none |
| **miR-106b** | 6,52 | 0,0006 | ST8SIA6 |
| **miR-143** | 46,64 | 0,0006 | TESC, ENTPD1 |
| **miR-22** | 30,56 | 0,0006 | C7 |
| **miR-19b** | 4,58 | 0,0006 | ITM2A |
| **miR-224** | 16,59 | 0,0007 | none |
| **miR-125b** | 22,62 | 0,0007 | none |
| **miR-24** | 9,89 | 0,0007 | none |
| **miR-222*** | 134,47 | 0,0008 | COL9A3 |
| **miR-362** | 14,83 | 0,0009 | none |
| **miR-374** | 7,52 | 0,0009 | none |
| **miR-517b** | 47,23 | 0,0010 | LTBP4 |
| **miR-10b** | 10,13 | 0,0010 | TESC, ITM2A |
| **miR-34a*** | 42,93 | 0,0010 | CCL19, SPP1 |
| **miR-27b** | 26,92 | 0,0011 | SPARCL1 |
| **miR-130a** | 10,49 | 0,0011 | none |
| **miR-376a** | 28,77 | 0,0011 | PPAP2A |
| **miR-27a** | 34,84 | 0,0012 | CCL19, SPARCL1 |
| **miR-589** | 9,28 | 0,0012 | none |
| **miR-193a-3p** | 237,84 | 0,0013 | none |
| **miR-210** | 99,97 | 0,0014 | COL9A3 |
| **miR-34c** | 15,94 | 0,0014 | none |

**Supplementary Table 5.** Differentially expressed genes found targeted by a high number of deregulated miRNAs

| mRNAs | FC.mRNA (Q vs. A) | miRNAs |
|---|---|---|
| ENTPD1 | 5,62 | miR-130b, miR-143, miR-23a, miR-27b*, miR-335*, miR-337-5p, miR-539, miR-654-3p, miR-708, miR-758 |
| SPARCL1 | 55,47 | miR-100, miR-140-3p, miR-152, miR-154*, miR-199a-3p, miR-19b-1*, miR-27a, miR-27b, miR-301b, miR-655 |
| VCAM1 | 13,98 | miR-132, miR-181a, miR-22*, miR-340, miR-376c, miR-424, miR-487a, miR-671-3p, miR-935 |
| ST8SIA6 | 14,01 | let-7i*, miR-106b, miR-184, miR-31, miR-31*, miR-365, miR-616, miR-98 |
| MTHFD1L | -9,43 | miR-126*, miR-142-5p, miR-200a, miR-429, miR-522, miR-605, miR-638 |
| PFKP | -16,73 | miR-139-3p, miR-139-5p, miR-142-5p, miR-372, miR-523, miR-548b-5p, miR-628-5p |
| ACOT7 | -4,97 | miR-139-3p, miR-200a, miR-200b, miR-302c*, miR-375, miR-429 |
| COL9A3 | 4,08 | let-7b*, miR-210, miR-222*, miR-31*, miR-597, miR-598 |
| DCBLD1 | -4,11 | miR-126, miR-139-3p, miR-302c*, miR-342-5p, miR-363, miR-885-5p |
| ITM2A | 10,63 | let-7a*, let-7b*, let-7i*, miR-10b, miR-19a, miR-19b |
| LOX | -67,33 | miR-126*, miR-200a, miR-200b, miR-429, miR-548b-5p, miR-628-5p |
| PPAP2A | 2,86 | let-7g, miR-100, miR-181a-2*, miR-18b, miR-376a, miR-503 |
| BTG2 | 23,21 | miR-100, miR-18b, miR-21, miR-31, miR-99a |
| CCL19 | 5,52 | miR-27a, miR-339-5p, miR-345, miR-34a*, miR-671-3p |
| DOCK7 | -8,94 | miR-126*, miR-135a, miR-142-5p, miR-223, miR-522 |
| FAP | -26,34 | miR-126, miR-135a, miR-200a, miR-375, miR-429 |
| SPP1 | 21,36 | miR-130b*, miR-181a, miR-34a*, miR-376c, miR-424 |
| TESC | 4,79 | miR-10a, miR-10b, miR-143, miR-339-5p, miR-532-3p |
| WNK4 | -7,06 | miR-135a, miR-142-3p, miR-202, miR-342-5p, miR-639 |

**FIGURE LEGENDS**

**Supplementary Figure 1: Integrative analysis pipeline**: **(A)** Differential microRNA and mRNA expression analysis. Heatmaps represent the 50 most significant miRNAs and mRNAs together with the volcano plots of the differential expressed miRNAs and mRNAs. Red and black dots represent significant (FDR<0.05) and non-significant differential expressed genes/miRNAs, respectively **(B)** Correlation and intersection with Microcosm Database. The table shows the basic statistics for correlations between miRNA and target genes. Only those miRNA-mRNA pairs with Pearson Correlation Coefficient < 0 and FDR<0.05 were considered and used for the intersection with Microcosm Database (8.7% of all potential correlations). Among all miRNA-target pairs predicted by MicroCosm (8463) only 9.16% present a negative and significant correlation and were considered for further analysis.

**Supplementary Figure 2: *In vitro* modulation of miRNA expression in human HSCs (LX2).** Reduction of miR-21 and miR-100 expression and up-regulation of miR-192 in LX2 cells was achieved by transfecting miR-21 antagomir (50nM), miR-100 antagomir (50nM) or miR-192 mimic (50nM), respectively (n=2) or control mimic/inhibitor. All expressions are relative to the solvent (blank).

# Supplementary figure 1

## A. Differential expression analysis

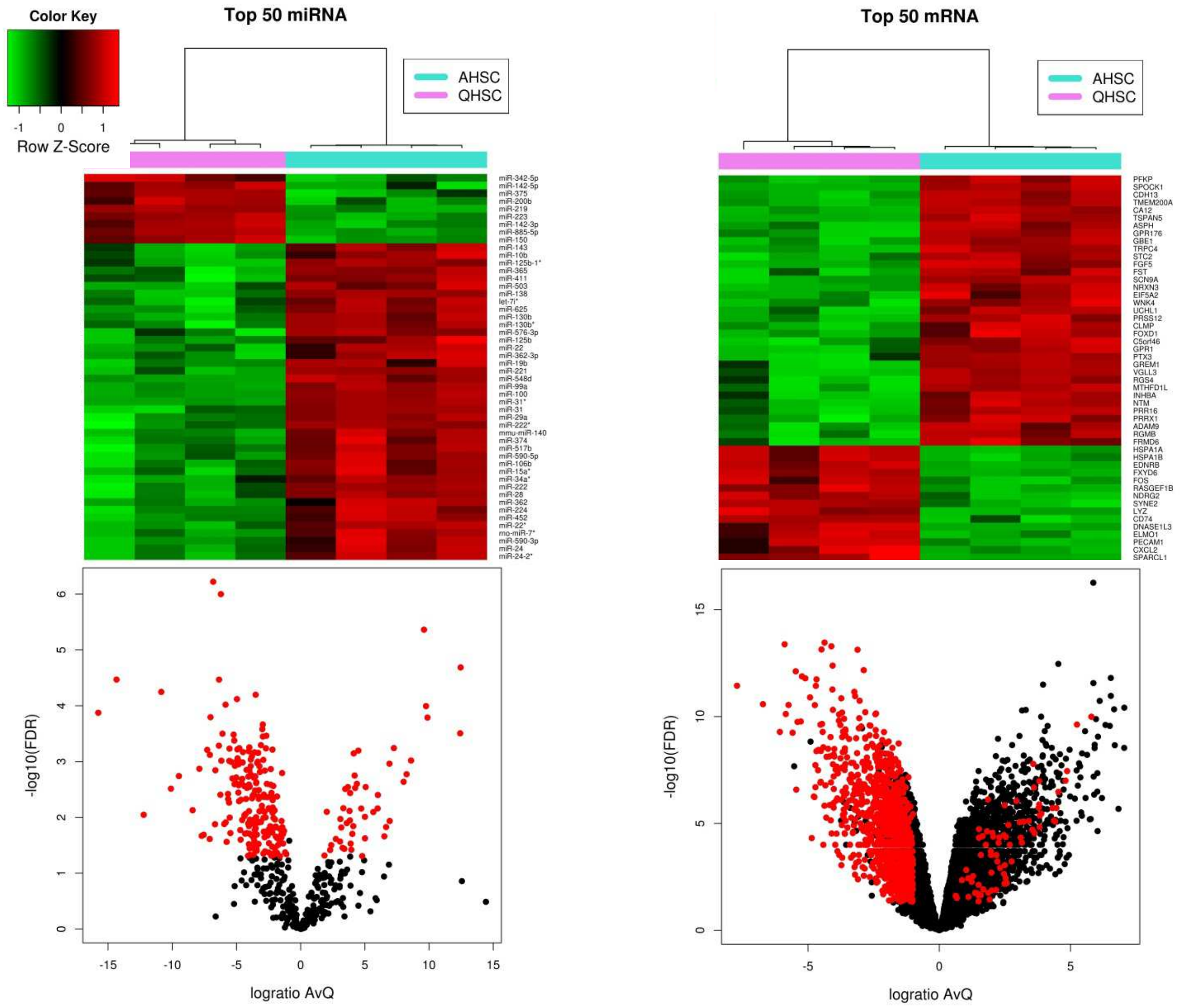


## B. Correlation and intersection with Microcosm Database

| | Number | % |
|---|---|---|
| Differentially expressed miRNAs | 259 | |
| Differentially expressed mRNAs | 1071 | |
| Number of total correlations | 276318 | 100 |
| Total negative correlations | 63032 | 22,81 |
| Total negative correlations, p<0,05 | 37130 | 13,43 |
| Total negative correlations, FDR<0,05 | 24031 | 8,70 |
| Total targets predicted in MicroCosm | 8463 | 100 |
| Total negative correlated targets | 1899 | 22,44 |
| Total negative correlated targets, p<0,05 | 1137 | 13,43 |
| Total negative correlated targets, FDR<0,05 | 775 | 9,16 |

# Supplementary Figure 2

**A.**

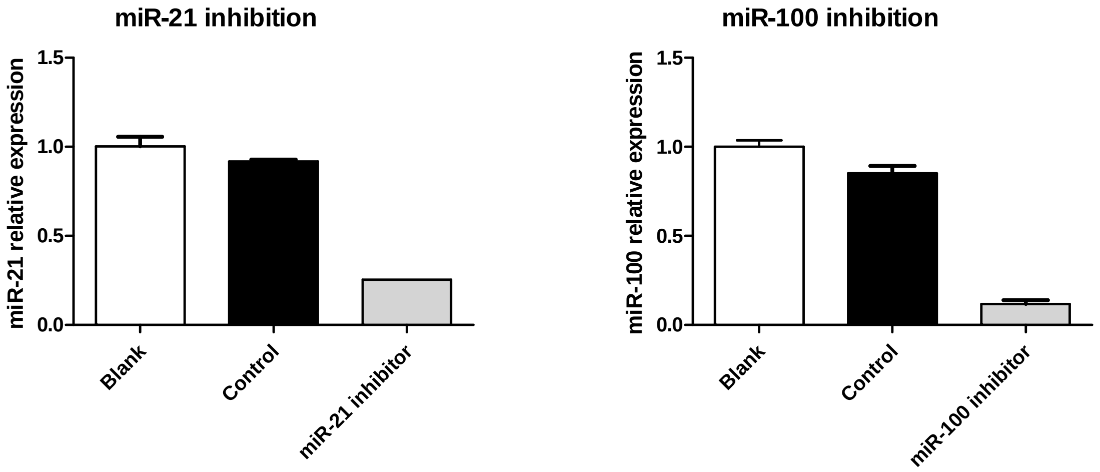


**B.**

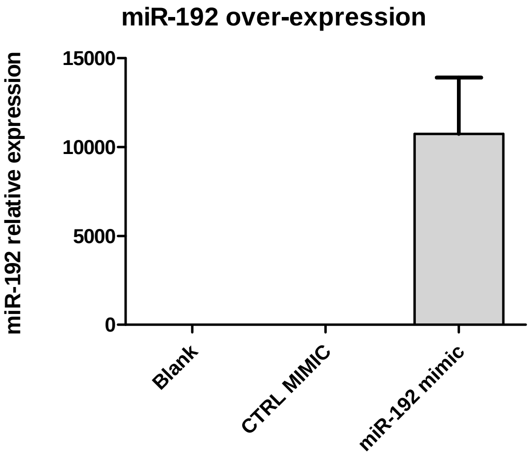

Supplement: Supplementary Information [file srep11549-s1.pdf]
